# Reconstitution of full‐thickness skin by microcolumn grafting

**DOI:** 10.1002/term.2174

**Published:** 2016-06-14

**Authors:** Joshua Tam, Ying Wang, Linh N. Vuong, Jeremy M. Fisher, William A. Farinelli, R. Rox Anderson

**Affiliations:** ^1^ Wellman Center for Photomedicine, Massachusetts General Hospital Boston MA USA; ^2^ Department of Dermatology Harvard Medical School Boston MA USA

**Keywords:** skin wound, full thickness, adnexa, healing, regeneration

## Abstract

In addition to providing a physical barrier, skin also serves a diverse range of physiological functions through different specialized resident cell types/structures, including melanocytes (pigmentation and protection against ultraviolet radiation), Langerhans cells (adaptive immunity), fibroblasts (maintaining extracellular matrix, paracrine regulation of keratinocytes), sweat glands (thermoregulation) and hair follicles (hair growth, sensation and a stem cell reservoir). Restoration of these functional elements has been a long‐standing challenge in efforts to engineer skin tissue, while autologous skin grafting is limited by the scarcity of donor site skin and morbidity caused by skin harvesting. We demonstrate an alternative approach of harvesting and then implanting μm‐scale, full‐thickness columns of human skin tissue, which can be removed from a donor site with minimal morbidity and no scarring. Fresh human skin microcolumns were used to reconstitute skin in wounds on immunodeficient mice. The restored skin recapitulated many key features of normal human skin tissue, including epidermal architecture, diverse skin cell populations, adnexal structures and sweat production in response to cholinergic stimulation. These promising preclinical results suggest that harvesting and grafting of microcolumns may be useful for reconstituting fully functional skin in human wounds, without donor site morbidity. © 2016 The Authors Journal of Tissue Engineering and Regenerative Medicine Published by John Wiley & Sons Ltd.

## 1 Introduction

Skin contains a complex architecture that is composed of many different cell populations and extracellular components, serving a remarkably diverse range of physiological functions, including physical barrier, sensation, innate and adaptive immunity, thermoregulation and protection from UV radiation. After skin is injured acutely by burns or trauma, or as a consequence of underlying pathologies in chronic wounds, the restoration of normal skin components/architecture is not a trivial undertaking – in fact, adult skin injuries with significant dermal involvement are generally unable to recover to the original unwounded state. Instead, they heal by scarring, which is characterized by disruptions in both epidermal and dermal architecture, prevalence of myofibroblasts, aberrations in the composition and organization of the extracellular matrix, and loss of skin appendages such as sweat glands and hair follicles. These faulty characteristics in turn lead to biomechanical defects, so that scars are stiffer and weaker than normal skin (Corr and Hart, 2013). In addition to abnormal structure, scars have abnormal metabolism and responses. Chronic symptoms include itching, contracture, tenderness and pain as well as psychosocial consequences, such as gross disfigurement, loss of self‐esteem, stigmatization, anxiety and depression (Monstrey *et al.*, 2014). Skin wounds and subsequent scarring represent an immense health care burden, annually affecting millions of people at the cost of tens of billions of dollars in the USA alone (Sen *et al.*, 2009).

Replacing lost skin tissue with skin harvested from autologous donor sites has been the mainstay of skin wound reconstruction since the nineteenth century (Davis, 1941) and remains so to this day (Buchanan *et al.*, 2014). Split‐thickness skin grafting (STSG), in which the epidermis and an upper portion of dermis are harvested from donor sites, then grafting onto wound sites, is currently the gold standard for closing large wounds (Brusselaers *et al.*, 2010). However, STSGs are associated with substantial drawbacks. A significant wound is created at the donor site, causing more pain, risk of infection, discolouration and scarring. The wound created at a donor site is frequently more problematic for patients than the primary wound being treated by STSG (Voineskos *et al.*, 2009; Demirtas *et al.*, 2010). Additionally, STSGs are usually meshed and expanded, causing a permanent and disfiguring 'fish‐net' appearance. Finally, STSGs include only the epidermis and upper dermis, lacking important deeper skin components, such as reticular dermal fibroblasts, hair follicles, sweat glands and other adnexal skin structures (Wong *et al.*, 2012). Consequently, the grafted tissues heal with scarring and are more prone to secondary contraction, desiccation and bagatelle trauma (Buchanan *et al.*, 2014). This last drawback in particular also applies to the various alternative approaches that are derived from STSGs, such as STSGs dispersed by mincing or enzymatic digestion, or expanded by *in vitro* culture.

Full‐thickness skin grafting, wherein the entire thickness of skin at a donor site is harvested, is currently the only therapeutic option capable of restoring the full complement of dermal components to a major wound site. Not surprisingly, skin wounds repaired using full‐thickness donor skin generally have the best post‐healing quality, with less scarring and secondary contraction (Buchanan *et al.*, 2014; Zhang and Meine, 2011). Interestingly, split‐thickness grafts are more prone to secondary contraction than full‐thickness grafts, even when the former is physically thicker than the latter (Harrison and MacNeil, 2008), which is yet another indication that the incorporation of cellular and/or extracellular elements in deeper parts of the skin (which are absent in split‐thickness skin grafts) is likely to improve the quality of wound healing. However, the improvements conferred by full‐thickness skin grafts come at the expense of fully sacrificing the donor site. Furthermore, full‐thickness skin grafts have higher metabolic demands than STSGs. If the graft does not rapidly receive a blood supply from the recipient wound, it dies. Reconstruction with full‐thickness skin is therefore primarily limited to smaller wounds in aesthetically important areas (such as the face), or in areas where secondary contraction would lead to more severe functional consequences (such as the hands) (Buchanan *et al.*, 2014).

Various forms of artificial skin substitutes have been developed in recent decades. These generally take the forms of decellularized skin tissue, extracellular matrix mimetics and cultured keratinocytes (Nyame *et al.*, 2014). While these options offer the great advantage of substantially reducing, or even completely eliminating, the need to harvest autologous donor tissue, they lack many of the cellular and extracellular components that make up natural skin (Eungdamrong *et al.*, 2014). The incorporation of diverse skin components has been a longstanding challenge in skin tissue engineering and, while there has been recent progress in incorporating additional skin components into bioengineered skin, e.g. melanocytes, hair follicles, and vasculature (Wu *et al.*, 2014; Marino *et al.*, 2014a; Swope *et al.*, 2002), it is not yet possible to recapitulate the full spectrum of skin components and structures using bioengineering techniques (Marino *et al.*, 2014b). Many of these techniques also rely on the use of allogeneic neonatal cell sources, which present additional immunological and regulatory hurdles for clinical translation. In addition, the complex and often labour‐intensive processes involved in producing skin substitutes add substantially to their cost. Due to these drawbacks, artificial skin substitutes have not yet been able to replace autologous skin grafting as the cornerstone of skin wound reconstruction.

There is one notable exception to the general rule that adult dermal wounds heal by scarring: very small wounds are able to heal by remodelling, i.e. complete restoration of normal skin features at both gross and microscopic levels. A common example is that passing a small steel hypodermic needle through skin causes a small wound that never scars, whereas passing a steel scalpel through skin causes a larger wound that always scars. This principle was previously utilized in our laboratory with the development of fractional photothermolysis (FP), a treatment that stimulates skin remodelling. FP uses laser microbeams to produce thousands of thin (hundreds of μm in diameter) columns of thermal burns per cm^2^ of skin surface. Each of these deep but small wounds heals rapidly, without scarring (Laubach *et al.*, 2006). The differences between FP and third‐degree skin burns are striking. Both involve extensive, full‐thickness thermal damage, but third‐degree burns heal slowly by scarring, whereas after FP the epidermis closes within hours and the dermal injury is repaired in a few weeks, followed by continued tissue remodelling without scarring (Laubach *et al.*, 2006). Impressively, up to 40% of the volume of normal skin can be killed or removed by FP, followed by rapid healing and regeneration, resulting in new skin tissue that is both functionally and aesthetically normal, and often improved (Laubach *et al.*, 2006). FP is now in widespread clinical use, both as a cosmetic treatment to 'rejuvenate' photoaged skin and also for scar revision (Saedi *et al.*, 2011; Anderson *et al.*, 2014). Interestingly, in scar tissue with (what was thought to be) permanent hair loss, FP treatment can lead to *de novo* hair regeneration (Beachkofsky *et al.*, 2011), which further underscores that healing in small skin wounds occurs through different mechanisms than in large skin wounds.

Based on this principle, that small skin wounds are capable of healing by remodelling and regeneration, we recently developed an alternative technique for harvesting full‐thickness skin tissue without causing donor site scarring. By harvesting skin in a multitude of μm‐scale full‐thickness skin columns, each donor wound could heal quickly by regeneration and without scarring. After harvesting, these skin columns were able to maintain viability and proliferative capacity *in vitro* (Rasmussen *et al.*, 2014). Using the swine model, we also found that this technique could be used to harvest a large number of full‐thickness skin columns without causing donor site scarring, and that the application of skin columns to full‐thickness skin wounds accelerated wound healing and re‐epithelialization (Tam *et al.*, 2013). However, it was not known whether grafting of full‐thickness microcolumns of human skin could transfer and recapitulate the complex structures of skin, such as hair follicles, sweat glands, pigmentation and immune system elements. The current study was performed in order to determine the feasibility of restoring adult human skin components *in vivo* using this technique, by evaluating the ability of these human‐derived skin components to persist, maintain expression of characteristic protein markers and recapitulate normal architecture and functions, following the engraftment of human skin columns in a murine wound model.

## 2 Materials and methods

### 2.1 Collection of human skin columns

Adult human abdominal skin tissue was obtained from elective abdominoplasties, with approval by the Massachusetts General Hospital Institutional Review Board. All human tissue samples used in this study were excess tissues that would otherwise be discarded, and were obtained without any patient information attached. Surfaces of the skin tissue were disinfected by soaking in a solution of 100 U/ml penicillin–streptomycin and 2.5 μg/ml Amphotericin B in normal saline, for 5 min. Columns of full‐thickness skin tissue were collected using a custom‐built harvester, consisting of a hypodermic needle modified to have two cutting edges (so that a piece of tissue is collected inside the needle bore with each insertion), coupled to a normal saline fluidic device that extracts and collects the skin columns using negative pressure, as described in detail previously (Tam *et al.*, 2013; Franco *et al.*, 2014). Harvesting needles of two different gauges (19 and 22 G, corresponding to inner diameters of approximately 700 and 400 μm, respectively) were used to collect full‐thickness skin columns of different diameters, to investigate the potential effects of column size on experimental outcome. Tissue viability in the harvested skin columns was assayed using the Live/Dead® Viability/Cytotoxicity Kit (Life Technologies), following the manufacturer's protocol.

### 2.2 Animal Procedures

All animal procedures were performed in accordance with the Public Health Service Policy on Humane Care and Use of Laboratory Animals, and approved by the Institutional Animal Care and Use Committee of the Massachusetts General Hospital. Adult female hairless mice with severe combined immunodeficiency (Crl:SHO*‐Prkdc* ^*scid*^ *Hr* ^*hr*^), purchased from Charles River Laboratories, were used for all experiments. The animals were anaesthetized by intraperitoneal injection of ketamine and xylazine (90/9 mg/kg). After disinfection with topically applied povidone–iodine, full‐thickness skin wounds of approximately 1 × 1 cm^2^ were produced by excision, and skin columns harvested from human abdominal skin were then applied directly into the wounds, to completely cover each wound (determined by visual inspection). The wounds were then protected by spraying on a layer of liquid barrier film (Cavilon, 3M), followed by Tegaderm film dressing (3M), and wrapping with an elastic bandage. This dressing regimen was maintained until an epidermal barrier became visible, generally 2–3 weeks after the original procedure, during which period the animals were inspected at least twice weekly, and dressings were changed when necessary. Thereafter, the dressings were removed and the wounds were monitored and periodically photographed.

### 2.3 Sweat testing

The ability of the reconstituted eccrine sweat glands to produce sweat was evaluated by an agonist‐induced sweat test, following the method described by Tafari *et al.* (1997), with minor modifications. Iodine was painted onto the skin region to be tested, allowed to dry, then followed by applying a coat of starch solution (5 g in 10 ml mineral oil); 200 μg acetylcholine was injected subcutaneously beneath the test region. Photographs taken before and 10 min after the administration of acetylcholine were compared to detect the presence of sweat, which could be visualized by the dark blue colouration that occurs when starch reacts with iodine.

The animals were euthanized 8 weeks after the implantation of human skin columns and the reconstituted skin tissue was collected for further analysis.

### 2.4 Histology and immunohistochemistry

Freshly excised tissue samples were fixed in 4% formaldehyde, embedded in paraffin, then sectioned at 5 μm and stained with haematoxylin and eosin (H&E) for histological evaluation. The expression of specific protein markers was investigated using immunohistochemical staining, as previously described (Tam *et al.*, 2013), with primary antibodies against various targets, as listed in Table 1. The antibodies against Ki67, PGP 9.5 and elastin stained both human and murine targets. All other antibodies were human‐specific and did not cross‐react with normal mouse tissue when processed with the same staining protocol (see supporting information, Figures S2–3).

**Table 1:** Antibody and dilutions for immunohistochemistry

Antibody	Vendor	Catalogue number	Dilution
CD1a	Abcam	ab108309	1:200
Elastin	Abcam	ab21610	1:100
Keratin 7	Abcam	ab68459	1:100
Keratin 8	Abcam	ab32357	1:50
Ki67	Abcam	ab16667	1:100
Major histocompatibility complex class I	Abcam	ab134189	1:200
Melan‐A	Abcam	ab51061	1:100
Nestin	Abcam	ab105389	1:100
PGP 9.5	Abcam	ab15503	1:4000
Vimentin	Vector Laboratories	VP‐RM17	1:100

## 3 Results

### 3.1 Human skin columns transferred to full‐thickness wounds in mice

**Figure 1:**
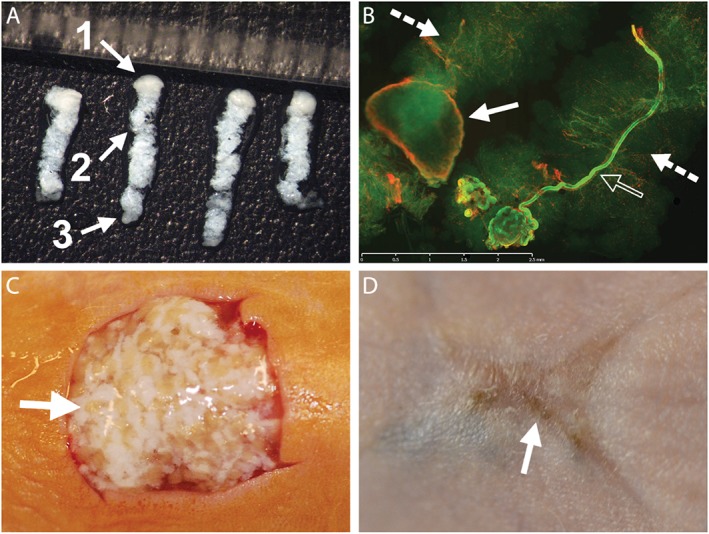
Harvesting and application of full‐thickness skin columns. (A) Full‐thickness skin columns harvested with a 19 gauge coring needle, each containing epidermis (1), dermis including adnexal structures (2) and some subcutaneous fat (3); each mark on the ruler in the photograph spans 1 mm. (B) Viability of skin columns after harvesting, demonstrated by calcein‐AM staining (green), with only a small portion of the tissue showing signs of cell death, as indicated by ethidium homodimer‐1 staining (red), mostly along the edge of the epidermis (solid arrow); one particular skin column contained a viable sweat gland (hollow arrow); dermal portions of the columns are highlighted by dashed arrows (for high‐power views of the epidermis and sweat gland, see supporting information, Figure S1); scale bar = 2.5 mm. (C) Human skin columns applied in random orientation to a full‐thickness wound on the dorsal skin of a mouse; arrow highlights the epidermal head of one skin column. (D) Photograph of the same wound site taken 8 weeks later; the wound has healed, with some pigmentation due to growth of the human epidermis (arrow). [Colour figure can be viewed at wileyonlinelibrary.com]

Using a custom‐made needle tissue harvester, many slender full‐thickness columns of skin tissue were collected from fresh adult human abdominal skin (obtained after abdominoplasty operations). Each column contained the epidermis, full dermis and a small amount of subcutaneous fat (Figure 1A). The diameter of each column could be controlled by varying the size of the harvesting needle. The experiments reported here were conducted using skin columns of either 700 or 400 μm in diameter, collected using 19 and 22 gauge harvesting needles, respectively. Each column size was repeated in eight animals. Similar results were obtained with the two column sizes, therefore data from both column sizes are presented in combination in this paper. The skin columns remained viable after harvesting (Figure 1B). The harvested skin columns were spread evenly into full‐thickness skin excision wounds on albino nude mice (Figure 1C) and the subsequent healing response was monitored. There was significant wound contraction, which is a typical wound‐healing response in mice. A new epithelium formed, which was pigmented (Figure 1D), notable because the animals were albino.

### 3.2 Recapitulation of epidermal skin components

**Figure 2:**
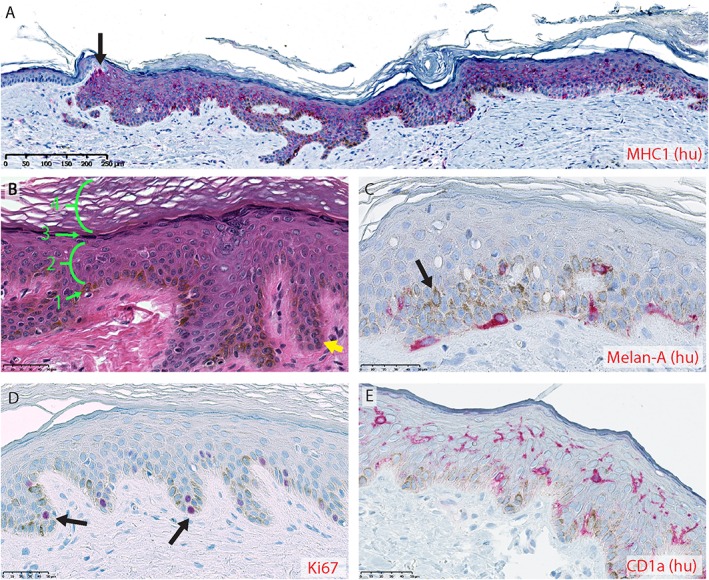
Restoration of human epidermal features, 8 weeks after murine full‐thickness wounds were repaired using human skin columns: positive immunohistochemical staining is shown in red; human‐specific staining is labelled as such by the suffix (hu) behind the name of the corresponding target. (A) Human keratinocytes, identified by positive staining for human major histocompatibility complex 1 (MHC1), forming a contiguous epidermis with an overlying human stratum corneum; arrow, transition point between murine (left, MHC1^–^) and human (right, MHC1^+^) epidermis at the wound margin. (B) Architecture of the restored epidermis, seen under H&E staining; all four layers of the epidermis, basal (1), spinous (2), granular (3) and cornified (4), are present in the correct ascending order; rete ridges are present at the dermal–epidermal junction (yellow arrow). (C) Melanocytes identified by Melan‐A expression (red) reside in the basal epidermal layer, while melanin is visible as brown pigmentation present in a perinuclear pattern in neighbouring cells (arrow). (D) Proliferative cells in the basal epidermal layer, identified by Ki67 expression (red, highlighted by arrows). (E) Dendritic Langerhans cells expressing CD1a (shown in red), populated most densely in the spinous layer of the epidermis. Scale bars = (A) 250 μm; (B, E) 50 μm. [Colour figure can be viewed at wileyonlinelibrary.com]

Eight weeks after the wounds had been treated with human skin columns, the restored skin samples were collected and analysed further using histology and immunohistochemistry. The treated wounds were re‐epithelialized with a contiguous epidermis composed of human keratinocytes, as shown by positive staining of the human‐specific marker MHC1 (Figure 2A). Furthermore, this epidermis recapitulated the normal architecture of human epidermis: it was stratified and organized into basal, spinous, granular and thick cornified layers, in ascending order, and contained prominent rete ridges at the dermal–epidermal junction (Figure 2B). Other human epidermal characteristics were also restored, including melanocytes (Figure 2C) and proliferative progenitors (Figure 2D) in the basal layer, as well as human Langerhans cells populating the spinous layer (Figure 2E). The melanocytes maintained their ability to produce and distribute melanin, which could be seen in the typical perinuclear intracellular pattern in neighbouring cells surrounding the melanocytes (Figure 2C).

### 3.3 Recapitulation of dermal skin components

**Figure 3:**
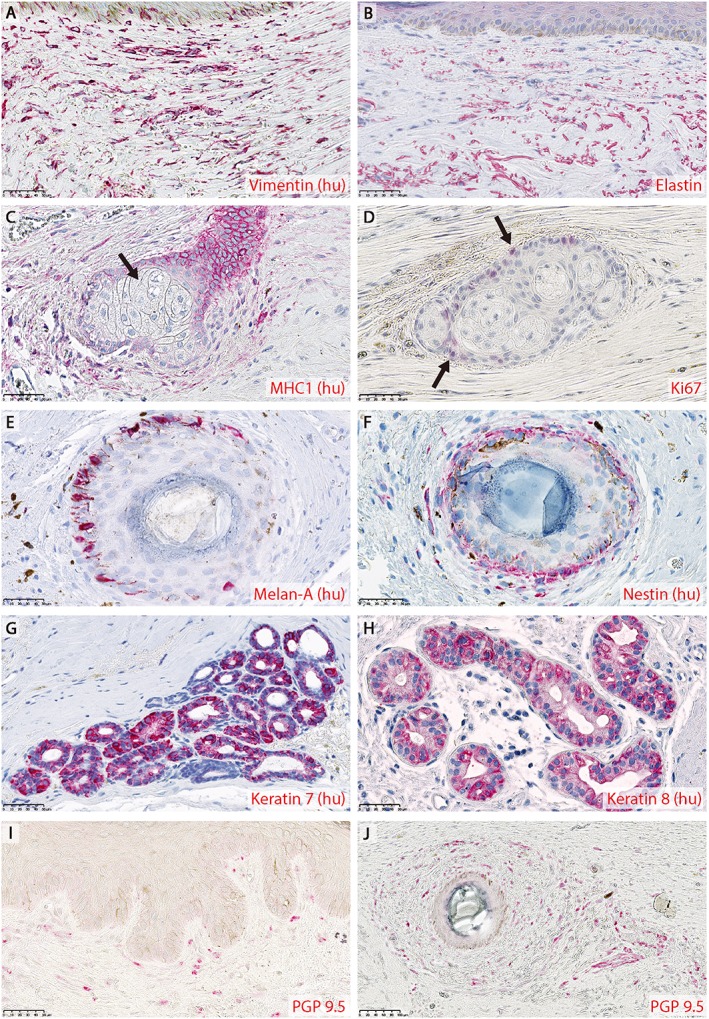
Restoration of human dermal features, 8 weeks after murine full‐thickness wounds were repaired using human skin columns: positive immunohistochemical staining is shown in red; human‐specific staining is labelled as such by the suffix (hu) behind the name of the corresponding target. Human fibroblasts expressing vimentin (A) and elastin (B) fibres were present throughout the restored dermis. Sebaceous glands of human origin, as shown by MHC1 staining (C), were also restored. Sebocytes containing lipid granules were present in the gland lumen (C, arrow). Proliferative cells, labelled by Ki67 (D, arrows), lined the periphery of the sebaceous glands. Hair follicles were associated with human melanocytes (E) and Nestin‐expressing progenitor cells (F). Eccrine sweat glands maintained the characteristic expression of cytokeratins 7 (G) and 8 (H). The restored dermis was innervated by PGP9.5‐expressing neurons through the dermis (I), but especially around the adnexal structures (J). Scale bars = 50 μm. [Colour figure can be viewed at wileyonlinelibrary.com]

Eight weeks after treatment, the skin restored with human skin columns also retained many human dermal skin components; fibroblasts expressing the human‐specific marker vimentin (Figure 3A) as well as elastin fibres (Figure 3B) that were dispersed throughout the reconstituted dermis. The elastin fibres were similar in thickness and structure to those in normal human skin (unlike scar tissue) and much thicker than those in normal mouse skin (see supporting information, Figure S3). Human sebaceous glands (Figure 3C) were present, with lipid‐laden sebocytes inside the gland lumen and proliferative precursors on the periphery of the gland (Figure 3D), thus recapitulating the normal cellular organization of human sebaceous glands, which is a central feature of the holocrine mechanism by which sebum is produced (Schneider and Paus, 2010). Hair follicles were present in the reconstructed skin and maintained their usual association with both melanocytes (Figure 3E) and nestin^+^ progenitor cells (Figure 3F). However, no hairs were visually observed on the reconstructed skin. Eccrine sweat glands, which do not normally exist in mouse skin outside of the paws, were present and maintained expression of the characteristic protein markers keratins 7 and 8 (Figure 3G, H, both human‐specific). The restored skin tissue was innervated with neurons expressing the neural marker PGP 9.5 throughout the dermis, but most densely around the adnexal structures (Figure 3I, J). The restored tissue was also vascularized, as clusters of red blood cells were present throughout the dermis, but only a small number of vessels expressed the human endothelial marker CD31 (not shown), suggesting that the reconstituted skin vasculature was primarily derived from the murine host.

### 3.4 Functional sweat glands

**Figure 4:**
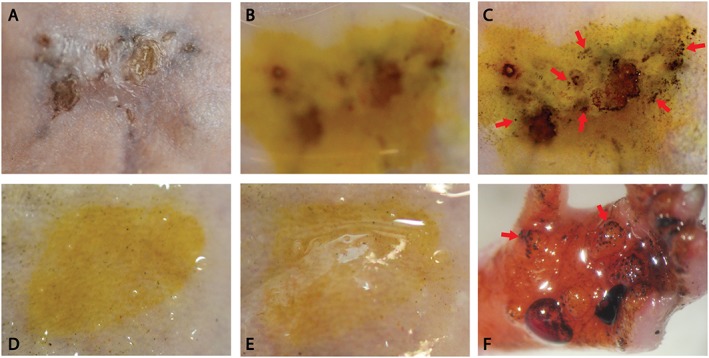
Agonist‐induced sweat test: 8 weeks after full‐thickness wounds treated with human skin columns (~400 μm diameter), the reconstituted skin (A) was covered first with iodine, then a starch–mineral oil mixture (B, blurriness of image caused by thick layer of starch‐mineral mix on top of the skin). Acetylcholine was injected subcutaneously under the region of interest: 10 min after acetylcholine was administered, sweat production could be detected by the formation of dark blue spots (C, arrows). As a negative control, normal mouse back skin, which is devoid of eccrine sweat glands, is shown before (D) and 10 min after (E) acetylcholine stimulation, with no detectable sweat production. As a positive control, the same test caused sweating in the normal mouse paw (F, arrows), which contains eccrine sweat glands. [Colour figure can be viewed at wileyonlinelibrary.com]

To determine whether the eccrine sweat glands in the reconstituted skin were actually able to produce sweat, we performed a modified form of the Minor test on four animals that were treated with the smaller (400 μm) columns. Iodine and starch were applied sequentially to the test sites, then acetylcholine was injected subcutaneously under the test sites to induce sweating. Sweating was detected when the sweat caused iodine and starch to mix, producing a characteristic deep blue colour. In three of the four animals tested, the reconstituted skin (all located on the dorsum of the animals) was able to sweat in response to acetylcholine stimulation (Figure 4A–C). As expected, normal mouse skin did not produce sweat under the same conditions (Figure 4D, E), except on the paws (Figure 4F).

## 4 Discussion

In this study we showed that small (400–700 μm diameter) columns of full‐thickness human skin tissue, harvested from adult abdominal skin, can be applied to wounds *in vivo* to restore many features of both epidermal and dermal skin. These features included a stratified epidermis with all four epidermal strata (corneum, granulosum, spinosum, and basale), appropriately located melanocytes and Langerhans cells, as well as rete ridges along the dermal–epidermal junction. Human dermal skin elements, including fibroblasts, neurons, elastin fibres and adnexal structures, also persisted for up to 8 weeks *in vivo*, and the restored sweat glands were able to produce sweat in response to acetylcholine stimulation. These results show that human skin microcolumns can be used to restore a broad range of skin features, which is currently only achievable with full‐thickness skin grafts. The restoration of functional sweat glands is a particularly exciting finding, as sweating is a crucial mechanism for thermoregulation in humans, and eccrine sweat glands contribute substantially to high‐quality wound healing (Rittie *et al.*, 2013). Unlike classical skin grafts, the skin microcolumns used in this study are within the small size range that can heal quickly and without scarring. This has been previously demonstrated in porcine models by us (Tam *et al.*, 2013) and others (Fernandes *et al.*, 2013), and also by the extensive non‐scarring clinical experience with fractional laser treatments (Manstein *et al.*, 2004; Saedi *et al.*, 2011).

Our technique of harvesting and grafting full‐thickness skin microcolumns was designed on purpose to be technically simple – it can easily be automated, and performed as a bed‐side procedure with routine local anaesthesia, without the need for exogenous cell sources, advanced biomaterials, *in vitro* culture, complex instrumentation or access to an operating room. These features minimize the regulatory and cost barriers involved with translating this technique into clinical use.

The practice of applying a multitude of small skin grafts to augment the healing of a larger wound dates back to at least 1869, when Reverdin developed the 'pinch grafting' technique, whereby a small section of skin was pinched, lifted up, then shaved off and grafted into wound sites (Ruiz Villaverde *et al.*, 2003). This did not include full‐thickness skin, however. Since then many alternative techniques have been developed to apply this concept, including mincing split‐thickness grafts into smaller pieces (Meek, 1958; Hackl *et al.*, 2012, 2014) and enzymatically digesting split‐thickness skin into single‐cell suspensions (Fraulin *et al.*, 1998; Navarro *et al.*, 2000). These techniques also fail to transfer full‐thickness skin structures, and lead to a donor site wounds that heal with scarring. Recently, our laboratory also developed a practical method to harvest arrays of epidermal suction microblisters, which can be applied to large wounds to enhance healing (Purschke *et al.*, 2015). Two main features distinguish the method described in this paper from the previous techniques mentioned above: (a) the ability to transfer full‐thickness skin tissue, particularly skin components found in deeper dermal regions, to wound sites; and (b) the μm‐scale donor wounds made by our method allow complete and scarless healing at the donor sites. To the best of our knowledge, there has only been one published study investigating the use of minced full‐thickness grafts, but that study did not address the engraftment/restoration of dermal skin components (Boggio *et al.*, 2008). The same group found that the *in vitro* release profiles of cytokines, chemokines and growth factors from full‐thickness skin were significantly altered by mincing, in a manner that may promote cell proliferation and angiogenesis (Pertusi *et al.*, 2012). This suggests that a similar phenomenon may be induced by the harvesting of skin microcolumns, a hypothesis that deserves further investigation.

Dermal fibroblasts are the major mesenchymal cell type in the dermis, and are responsible for constructing and maintaining the extracellular matrix, which in turn determines much of the skin's mechanical properties. Rheinwald and Green (1975), in their seminal work on the serial culture of human keratinocytes, established that fibroblasts also play a key role in maintaining epidermal function. For the purposes of skin tissue engineering, dermal fibroblasts were traditionally considered a rather replaceable cell type. Following Rheinwald and Green, replication‐inactivated fibroblasts derived from allogeneic (e.g. human neonatal foreskin) or xenogeneic (e.g. murine embryonic 3T3 cells) sources are commonly used to produce bilayered bioengineered skin constructs (Rasmussen *et al.*, 2013; Wong *et al.*, 2007), and mesenchymal cells from various other tissues have also been used to replace dermal fibroblasts for a similar supportive role in skin engineering (Bottcher‐Haberzeth *et al.*, 2014; Hartmann‐Fritsch *et al.*, 2013). However, recent studies have shown that dermal fibroblasts are a far more complex cell type than previously appreciated. Dermal fibroblasts consist of diverse subpopulations arising from different lineages, and vary significantly between different anatomical locations, as well as between different skin depths (Sorrell and Caplan, 2009; Janson *et al.*, 2012; Driskell *et al.*, 2013). Paracrine signals from local dermal fibroblasts are responsible for maintaining the specialized epidermal characteristics of skin in different anatomical locations, such as the thickened, load‐bearing skin in palms and soles (Yamaguchi *et al.*, 2005). In murine skin wounds, fibroblasts from the upper dermis are required for hair follicle formation, whereas fibroblasts from the lower dermis are largely responsible for constructing the extracellular matrix, and the imbalance between these fibroblast subpopulations in skin wounds is at least partially responsible for the fibrosis and lack of adnexal structures in skin scars (Driskell *et al.*, 2013). Importantly for tissue‐engineering applications, some of these distinctive phenotypes are lost after extended *in vitro* culture (Janson *et al.*, 2013). Given this new understanding, it seems reasonable to postulate that the inclusion of fresh (i.e. non‐cultured) autologous dermal fibroblasts from all layers of skin, which is enabled by our technique, is likely to have beneficial effects on wound healing, particularly on the biomechanical properties of the post‐healing skin. We plan to address this aspect of our technique in future studies in larger animal models and, ultimately, in clinical trials.

One of the main drawbacks of our method is that barrier function is not immediately restored, similar to other techniques using minced/digested tissue for grafting. Therefore, barrier function would have to be provided by some other means during the early post‐treatment period. Fortunately, there are many existing clinical options for providing temporary barrier function, from various bandages and dressings to advanced biomaterials. In particular, dressing regimens that maintain a moist wound environment have been shown to enable the engraftment of split‐thickness skin grafts with no detrimental effects, even when the grafts were placed upside‐down, as keratinocytes are able to migrate from the base of the wound up to the surface, where they then participate in wound re‐epithelialization (Hackl *et al.*, 2012, 2014; Zuhaili *et al.*, 2010). This is salient because the skin columns in our study reported here were also applied randomly into wounds, without maintaining the epidermis‐up orientation of normal skin.

The functional human adnexal structures (such as sweat glands and hair follicles) observed after microcolumn grafting in this study may be a subpopulation that survived being transferred, and/or structures that were newly generated during the wound‐healing process. Adnexal structures are home to stem and progenitor cell populations that are able to regenerate the corresponding structures *de novo* (Lu *et al.*, 2012; Wu *et al.*, 2014). In particular, neogenesis of hair follicles occurs during the healing of full‐thickness wounds in mice (Ito *et al.*, 2007), suggesting that new human hair follicles could also be induced. It also remains unclear whether the dermal adnexal structures within skin microcolumns are able to reorientate after grafting, similar to the epidermis. Potentially, the survival or neogenesis of these complex structures may be improved by maintaining the normal skin orientation when applying the skin microcolumns to a wound bed.

Limitations of this study include the use of human‐to‐murine rather than human‐to‐self autologous skin microcolumn grafts. The influence of body site remains unknown, e.g. whether palmoplantar skin retains its special characteristics after this form of grafting. In this study, nothing other than the grafted skin columns was used for grafting. Although in this study we have focused on the most straightforward strategy for applying skin columns, i.e. placing the columns randomly into wounds without any exogenous biomaterials, the skin columns could potentially be combined with engineered tissue materials, cells, drugs, growth factors, etc., to achieve synergistic effects. For example, an array of skin microcolumns, properly orientated, could be implanted into a dermis‐like matrix material that supports outgrowth of cells from each column. Our technique for collecting autologous skin tissue could also be useful for ‘traditional’ tissue‐engineering methods, by providing an autologous cell source with minimal donor site morbidities.

### Conflicts of interest

*J.T.*, *Y.W.*, *W.F. and R.R.A. are co‐inventors in patent applications filed from the Massachusetts General Hospital*, *based on the technology described in this manuscript*, *and hold co‐founder equity in a company recently founded to develop and commercialize this technology. The company had no involvement in this study*, *which was not supported by any commercial entity.*

## Supporting information

Figure S1. High‐power views of live/dead staining in skin columns
Figure S2. IHC staining in normal human and murine skin tissues
Figure S3. Antibodies with cross‐reactions in normal human and murine skin tissues

Supporting info tem
