# Supplementary figures and images for: Reconstitution of full‐thickness skin by microcolumn grafting
Source: J Tissue Eng Regen Med. 2016 Jun 14;11(10):2796–805. doi: 10.1002/term.2174 (PMC5697650; doi:10.1002/term.2174)

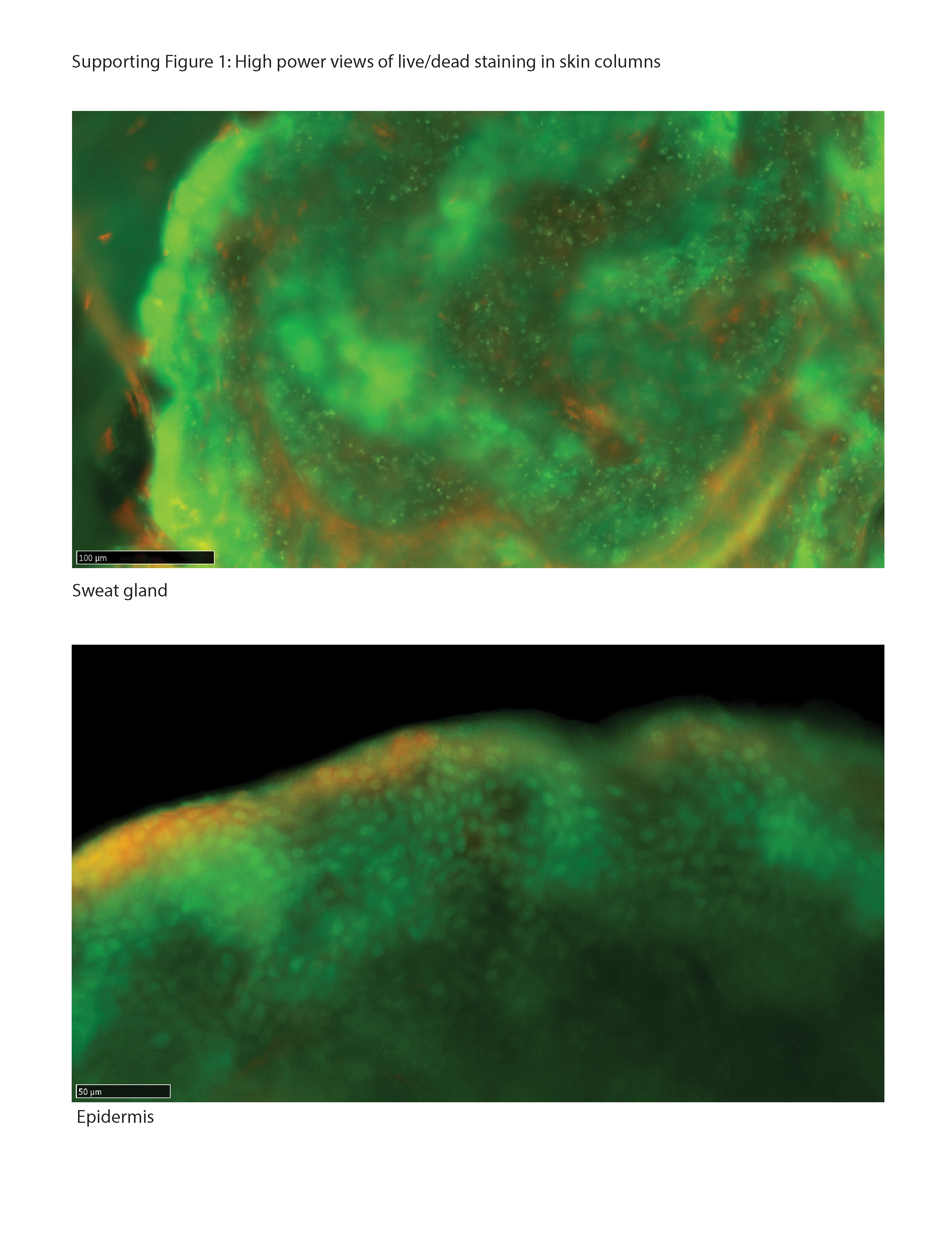

Supplement: Supplementary file 1 — Supporting info tem [file TERM-11-2796-s001.zip › Supporting Information 1.png]

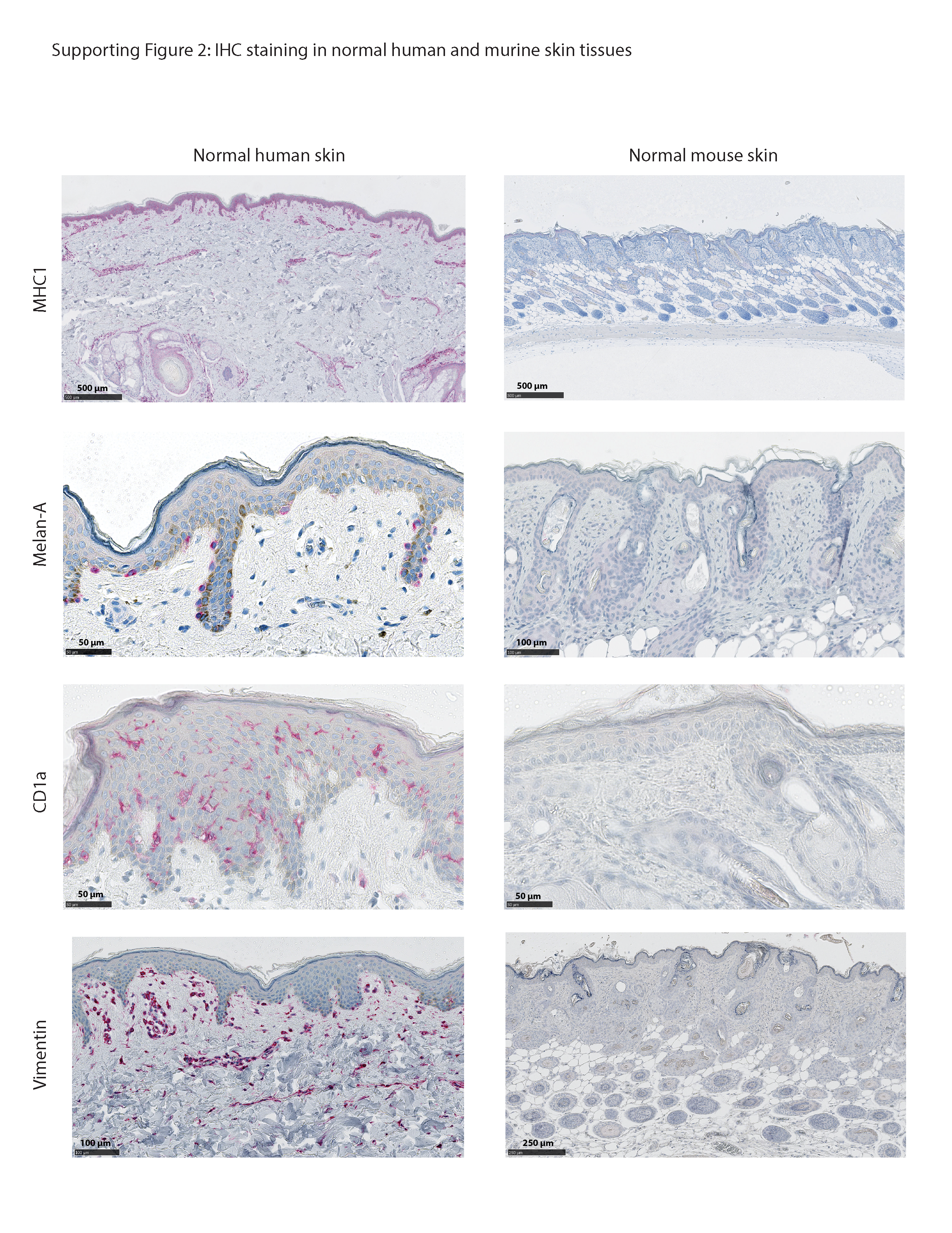

Supplement: Supplementary file 1 — Supporting info tem [file TERM-11-2796-s001.zip › Supporting Information 2.png]

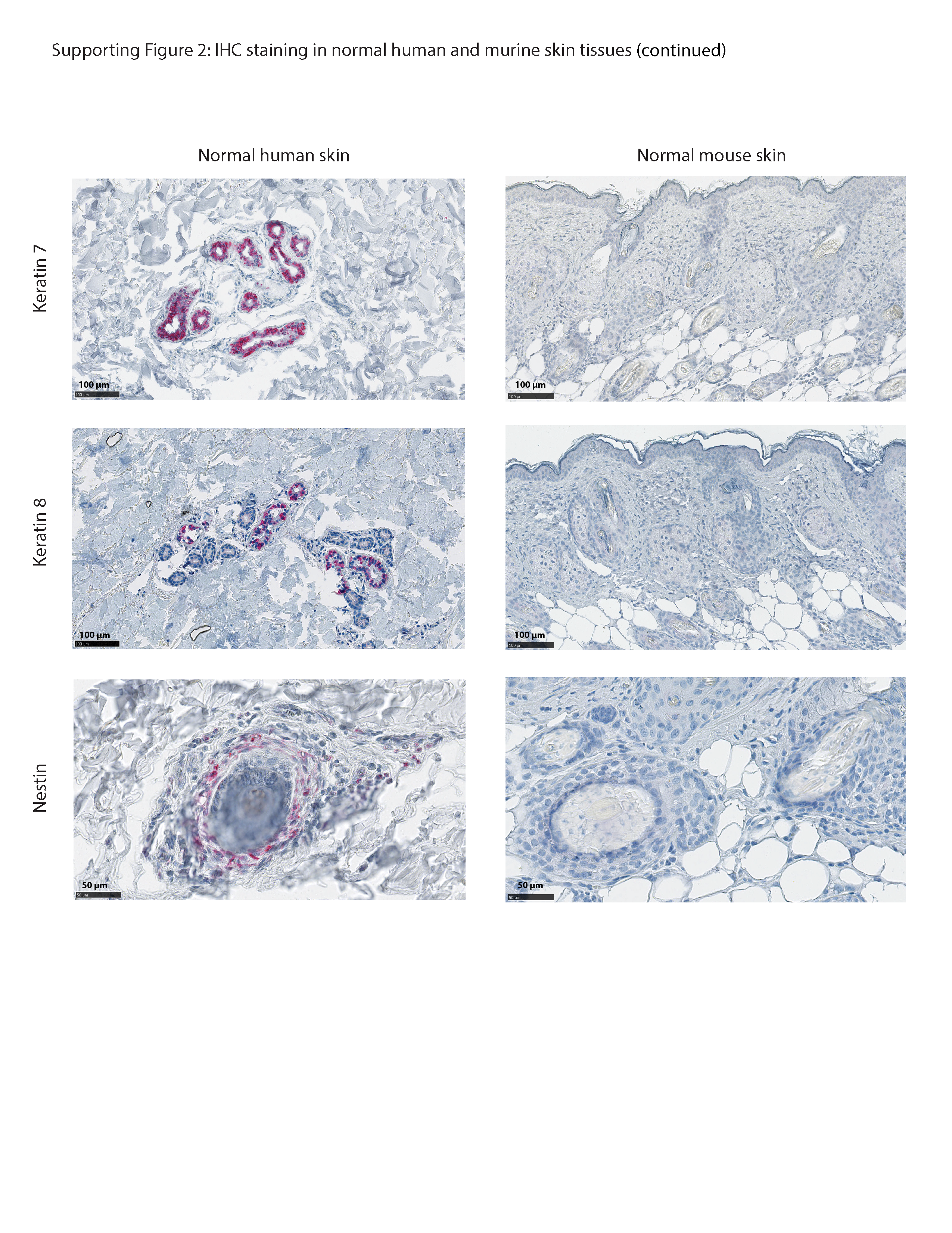

Supplement: Supplementary file 1 — Supporting info tem [file TERM-11-2796-s001.zip › Supporting Information 3.png]

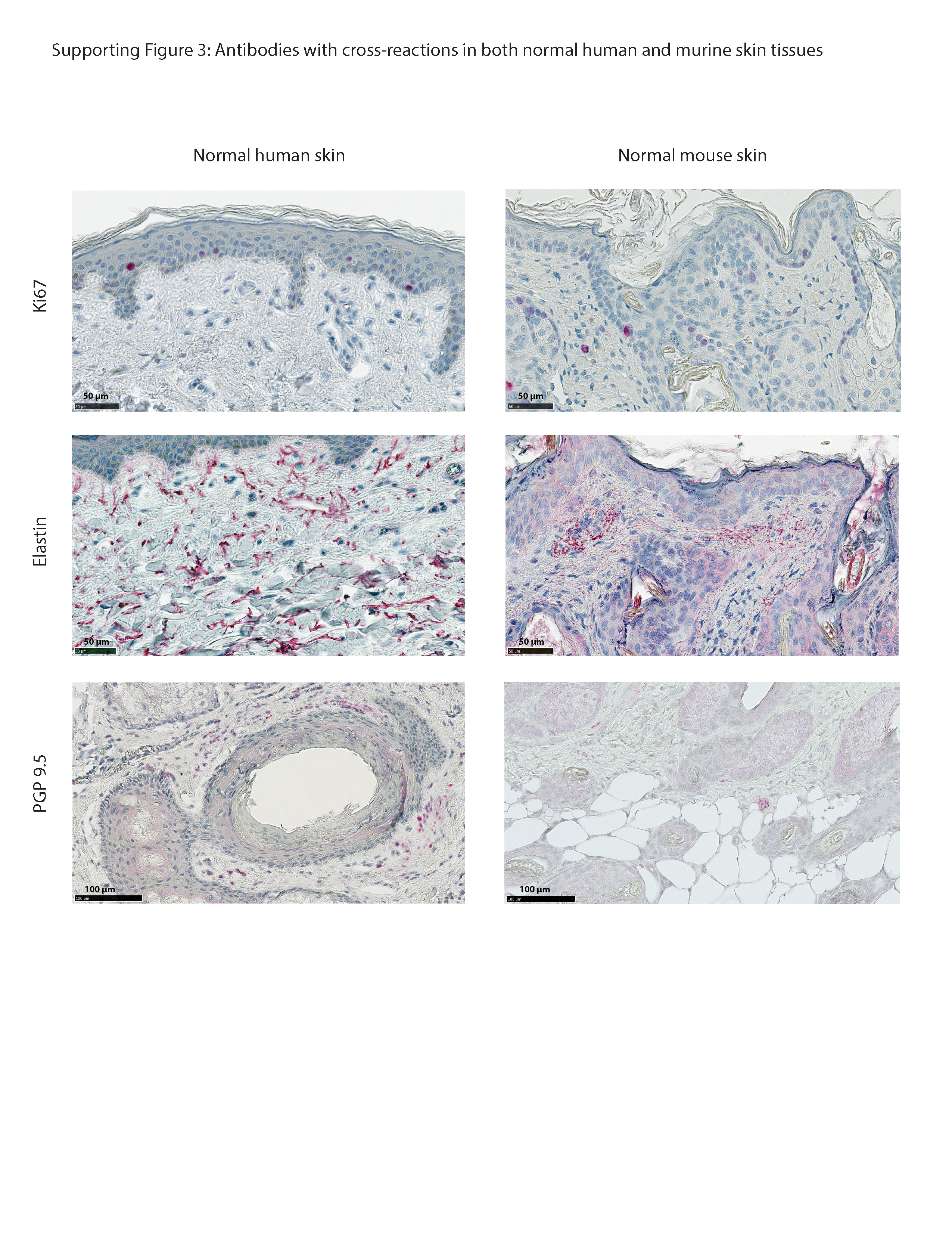

Supplement: Supplementary file 1 — Supporting info tem [file TERM-11-2796-s001.zip › Supporting Information 4.png]
